# Nitrite manipulation in water by structure change of plasma electrolysis reactor

**DOI:** 10.1038/s41598-024-75046-4

**Published:** 2024-10-05

**Authors:** Fatemeh Baharlounezhad, Mohammad Ali Mohammadi

**Affiliations:** 1 Faculty of Physics, University of Tabriz, Tabriz, Iran; 2 Research Institute of Applied Physics & Astronomy, University of Tabriz, Tabriz, Iran

**Keywords:** Ammonium, Electrolysis, Nitrification, Nitrate, Nitrite, Nitrogen, Plasma, Water, Physics, Electrochemistry

## Abstract

In this study, experimental reactors for cathodic nitrogen plasma electrolysis were designed by the composition of galvanic (voltaic) and electrolytic cells with wide and narrow connectors filled with tap water and agar solutions. The designed reactor can be used to simultaneously perform and manage nitrification in acidic and alkaline environments. According to the reactor’s performance, it can be installed on the irrigation system and used depending on the soil pH of the fields for delivering water and nitrogen species that are effective in growth. The nitrification process was investigated by choosing the optimal reactor with a wide connector based on different changes in oxidation-reduction potential and pH on the anode and cathode sides. The nitrite concentration changed directly with ammonium and nitrate concentrations on the cathode side. It changed inversely and directly with ammonium and nitrate concentrations on the anode side respectively. Nitrite concentration decreased from 5.387 ppm with water connector, to 0.326 ppm with 20% agar solution, and 0.314 ppm with 30% agar solution connectors on the anode side. It increased from 0 ppm to 0.191 ppm with a water connector, 0.405 ppm with 20% agar solution, and 7.454 ppm with 30% agar solution connectors on the cathode side.

## Introduction

Microbial activities normally control the exchange between atmospheric inert nitrogen gas and reactive nitrogen. The nitrogen cycle as a biogeochemical procedure converts the inert nitrogen of the atmosphere into a more operational factor for living organisms. Nitrogen is fixed in the nitrogen cycle through biological, atmospheric, or industrial processes. Nitrification as a part of the nitrogen cycle is a two-step aerobic process by which naturally microorganisms oxidize ammonia or ammonium $\:(\text{N}{\text{H}}_{3}\:/\text{N}\:{\text{H}}_{4}^{+})$ to nitrite $\:\left(\text{N}{\text{O}}_{2}^{-}\right)$ and then nitrate $\:\left(\text{N}{\text{O}}_{3}^{-}\right)$^1–3^. The opinion of ammonia oxidation to nitrate as a biological process was first presented by Louis Pasteur^4^. Nitrification is widely applied in water purification to eliminate nitrogen from municipal wastewater^5^. Nitrogen in nitrate form is broadly used as fertilizer. Nitrification plays an essential role in agricultural systems to convert ammonia via nitrite to nitrate which is effective in growing plants^6,7^.

Non-thermal plasma (NTP), known as cold plasma, has an electron temperature much higher than positive ions and neutral particles. Cold atmospheric plasma technology is an eco-friendly technology and water purification using electrical energy is one of its growing potential applications. This technique is an eco-friendly, simple, and effective method for decomposing toxic organic compounds in raw water or wastewater and has been developed since 1987. Nowadays, research is increasingly done to purify water using atmospheric plasma because it is a useful method that does not require adding other chemical agents^8–15^. Cold plasma also contributes to agriculture focused on the different applications of plasma technology in various crop production steps. It increasingly performs as a green alternative to conventional agrochemical treatments which includes plasma treatment of liquid solutions to remove contaminant chemical compounds and plasma-activated water (PAW) utilization to improve plant growth^16,17^.

Electrolysis is the passing process of an electric current through a substance to cause a chemical change. This process occurs in reactor cells consisting of cathode and anode electrodes immersed in a solution containing positively and negatively charged ions. Electrolysis is widely used in metallurgical processes^18,19^, such as electrowinning^20,21^, electrorefining^22–24^, and electroplating^25,26^. There are two types of galvanic (voltaic) and electrolytic cells with many differences in general. Some of the differences are mentioned in Table 1. The discharge phenomena related to electrolysis were discovered more than a century ago by Sluginov and were discussed in detail in the 1930s by Günterschultze and Betz^27,28^.

**Table 1:** Differences between galvanic and electrolytic cells.

Electrolytic cell	Galvanic cell
It uses an electric current to create a chemical reaction.	It is an electrochemical cell that can produce electricity using a chemical reaction.
It converts electrical energy into chemical energy.	It converts chemical energy into electrical energy.
The anode is positively charged, and the cathode is negatively charged.	The anode is negatively charged, and the cathode is positively charged.
A reaction occurs non-spontaneous.	A reaction occurs spontaneously.
The reduction happens in the cathode, and oxidation occurs in the anode.	The oxidation occurs at the anode, and reduction happens at the cathode.
Electrical energy produces a chemical reaction with an external source.	Electrical energy is produced by chemical reactions.
Electrodes are placed in a container containing molten or liquid electrolytes (in some cases, they are placed in two containers connected by a salt bridge).	The half-cells are placed in a separate container and are connected through the connector (salt bridge).

Nitrogen (N) is a key element for the growth of all plants and is counted as the most extensively used agricultural fertilizer nutrient in the world. About half of the global N fertilizer used in agriculture systems is not absorbed by plants^29^. It is lost into the environment as nitrogen species like ammonia, nitrite, and nitrate through different ways, causing an environmental crisis and increasing costs^30–32^. Ammonia negatively affects plant, biotic, and abiotic components of the environment; few plants can conveniently use ammonia^33,34^, and nitrate is most sensitive to leaching and an abundance of it also reduces the demand for nitrate^35^; Therefore, a gradual release is needed at the right time. Under certain circumstances, controlling nitrogen species can have a helpful impact on drinking water quality in the controlled conditions at a water treatment plant. Due to the detrimental effects, inhibitors have recently been used to restrict and control the nitrification rate^36,37^. Selective production methods of ammonium, nitrite, and nitrate can be advantageous in agriculture to achieve more crop yield. As an evident intermediate product in the nitrification process, nitrite has been reported as effective in the efficiency of ammonia oxidation and nitrate production^38^. Several researchers have reported methods to control the production of nitrogen species through plasma^39,40^and plasma-assisted nitrification^41^, but until now, the concurrent ambivalent attitude of controlling nitrogen fixation and nitrification by plasma-water interaction has not been discussed.

Here, a green method was discussed to reduce nitrogen fertilizer loss to the environment, optimize nitrification efficiency, and increase nitrogen use efficiency in crops via plasma electrolysis without any chemical usage. The common nitrification rate in soil, sediments, and aquatic environments is determined by environmental factors such as water potential, temperature, oxygen, and pH. We used the cathodic cold atmospheric plasma electrolysis in a galvanic and electrolytic cells combination to evaluate water based on oxidation-reduction (redox) potential (ORP) changes for the plasma nitrification process. The ORP of tap water was determined after plasma exertion at various times and reactors. According to the ORP results, the optimal reactor was chosen to perform and investigate the nitrification process via plasma electrolysis. The mechanism and chemical reactions at the first step of plasma electrolysis leading to nitrification were described. The concentrations of ammonium, nitrite, and nitrate ions resulting from nitrogen fixation and nitrification in the optimal reactor were determined using ion chromatography. Plasma electron density was measured by the Stark broadening of the hydrogen Balmer $\:{\text{H}}_{\beta\:}$ spectral line. Plasma electron temperature was determined by the Boltzmann plot using the plasma emission spectroscopy of glow discharge.

## Materials and methods

**Fig. 1:**
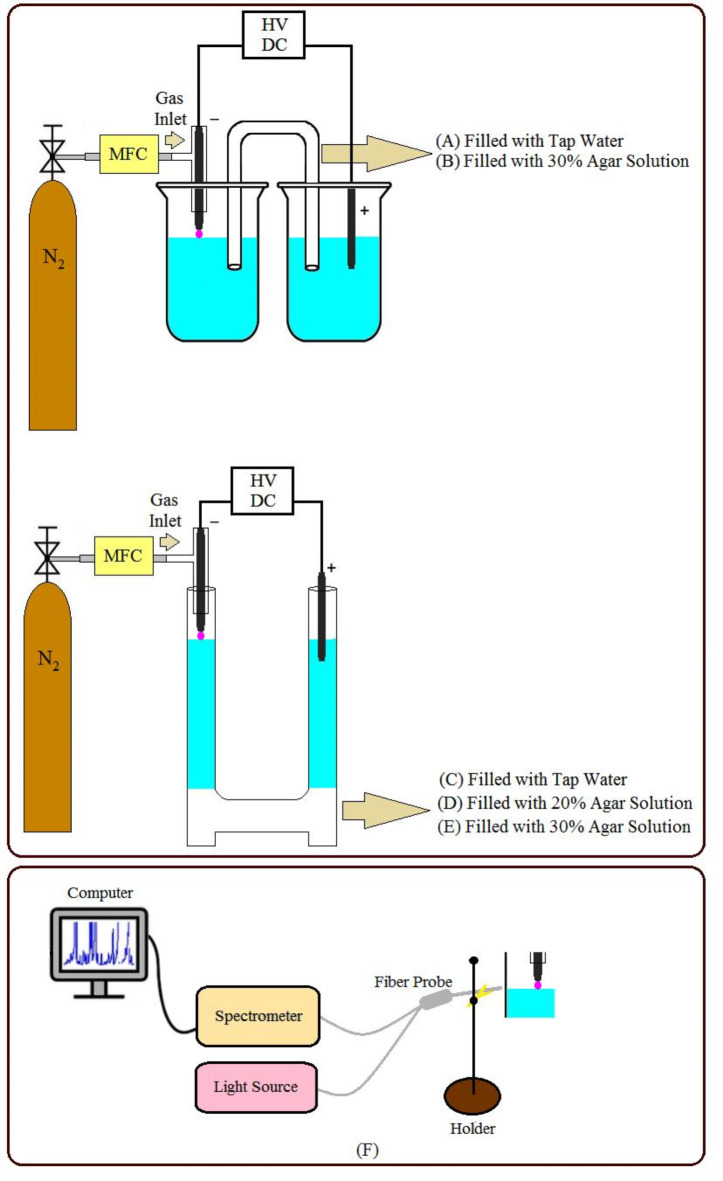
Experimental setup (Cathodic plasma electrolysis experimental reactor with a narrow Pyrex connector filled with (**A**) tap water and (**B**) agar solution, and with a wide Pyrex connector filled with (**C**) tap water, (**D**) 20% agar solution, and (**E**) 30% agar solution, and (**F**) miniature optical spectrometer).

Figure 1 shows experimental reactors for cathodic plasma electrolysis through the composition of galvanic and electrolytic cells. A Pyrex Hoffman electrolysis reactor was used with a Pyrex connector to separate the anode and cathode liquid and to permeate ions for nitrification in the water. According to Fig. 1, the narrow Pyrex connector (inner diameter 5 mm) was filled with (A): tap water and (B): 30% agar solution. The wide Pyrex connector (inner diameter 30 mm) was also filled with (C): tap water and (D, E): agar solution. Agar solution was prepared with (D): 20% and (E): 30% of mass (g)/volume (mL) percent, respectively. Reactor-filling tap water was the same in all cases (pH = 6.68, EC = 114 µS/cm). The electrodes were cylindrical tungsten rods with an external diameter of 1.5 mm. The tip of the cathode electrode sitting inside the glass tube was placed 5 mm above the water and the anode electrode was located inside the water. The reactors were filled with 200 ml of tap water at room temperature (after solidifying the agar solution in types B, D, and E). Plasma was produced by nitrogen gas with a DC high-voltage power supply between the cathode electrode tip and the water surface at 7 kV. The gas flow rate was set at 40 sccm. The experiments were done in 0–6 min during the 0.5 min periods in any reactors.

Each experiment was repeated five times. The reported values (ORP, pH, and ion concentration) were the average values of five repetitions in each type of reactor at a certain plasma-exerting time. Water pH and ORP were measured with the pH/ORP meter (HI2002 - edge^®^ Dedicated pH/ORP Meter) of HANNA company with a numerical precision of 0.01 and a five-point buffer calibration before measurement. Ions concentration (nitrite, nitrate, and ammonium) measurement was done by ion chromatography (930 Compact IC Flex, 150 mm version of the Metrosep A Supp 5–150/4.0) made by Metrohm company with a particle size of 5 μm. IC system was calibrated by creating a calibration curve with five calibration solutions. The emission spectrum of nitrogen plasma was recorded with a miniature optical spectrometer (UV-Vis-NIR), with a wavelength accuracy of ± 0.1 nm, manufactured by Pooyesh Tadbir Karaneh Co. Spectrometer calibration was performed by removing background noises and recording the spectrum of the reference source with 50 ms spectrum collection time and 5 the average number of spectrum collections.

## Results

### Plasma parameters

The plasma electron temperature was specified using the standard Boltzmann plot technique by choosing four proper lines of (N I) from nitrogen spectral line emission intensity and the following equation^42^.

$$\:\text{l}\text{n}\frac{{\text{I}}_{\text{j}\text{i}}{{\uplambda\:}}_{\text{j}\text{i}}\left(\text{n}\text{m}\right)\:}{{\text{A}}_{\text{j}\text{i}}{\left({\text{s}}^{-1}\right)\text{g}}_{\text{j}}}=-\frac{{\text{E}}_{\text{j}}\left(\text{e}\text{V}\right)}{{\text{k}}_{\text{B}}(\text{J}/\text{K})\text{T}\left(\text{e}\text{V}\right)}+\text{c}\:\:\:\:\:\:\:\:\:\:\:\:\:\:\:\:\:\:\:\:\:\:\:\:\:\:\:\:\:\:\:\:\:\:\:\:\:\:\:\:\:\:\:\:\:\:\:\:\:\:\:\:\:\:\:\:\:\:\:\:\:\:\:\:\:\:\:\:\:\:\:\:\:\:\:\:\:\left(1\right)$$

Atomic data of selected N I lines were displayed in Table 2^43,44^.

**Table 2:** Atomic data of N I lines.

Wavelength(nm)	Transition probability$\:\left({10}^{8}{\mathbf{s}}^{-1}\right)$	Statistical weight	Upper level energy (eV)
439.24	0.0102	2	14.9483
536.70	0.0012	4	13.2388
537.26	0.0011	4	13.2364
585.60	0.0076	2	13.9563

**Fig. 2:**
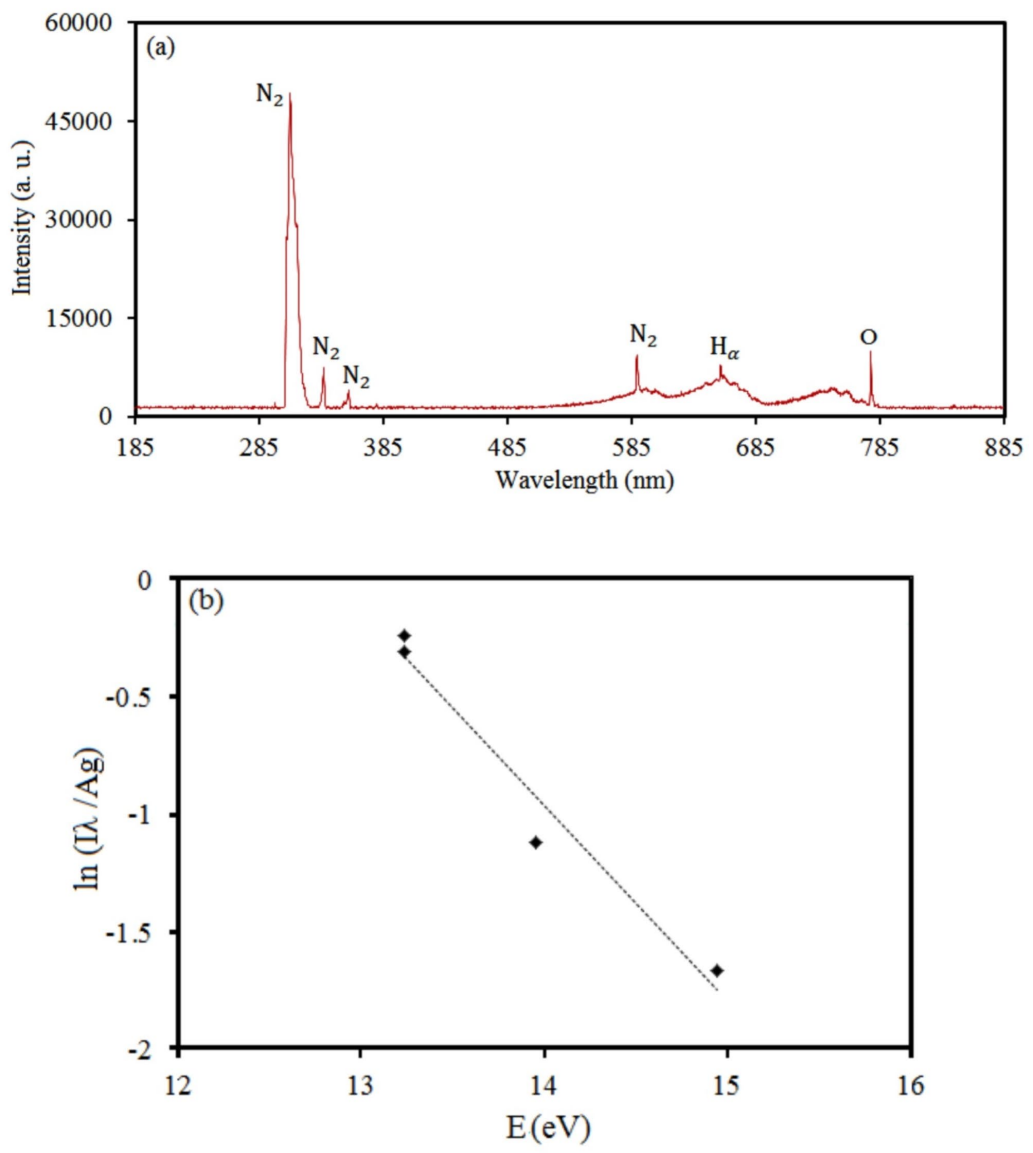
(**a**) Spectra of nitrogen plasma emission and (**b**) the Boltzmann plot of N I spectral lines at 7 kV.

Figure 2 is the spectra of nitrogen plasma emission and the Boltzmann plot for calculating plasma electron temperature at 7 kV. The plasma electron temperature was calculated at 0.8274 eV.

**Fig. 3:**
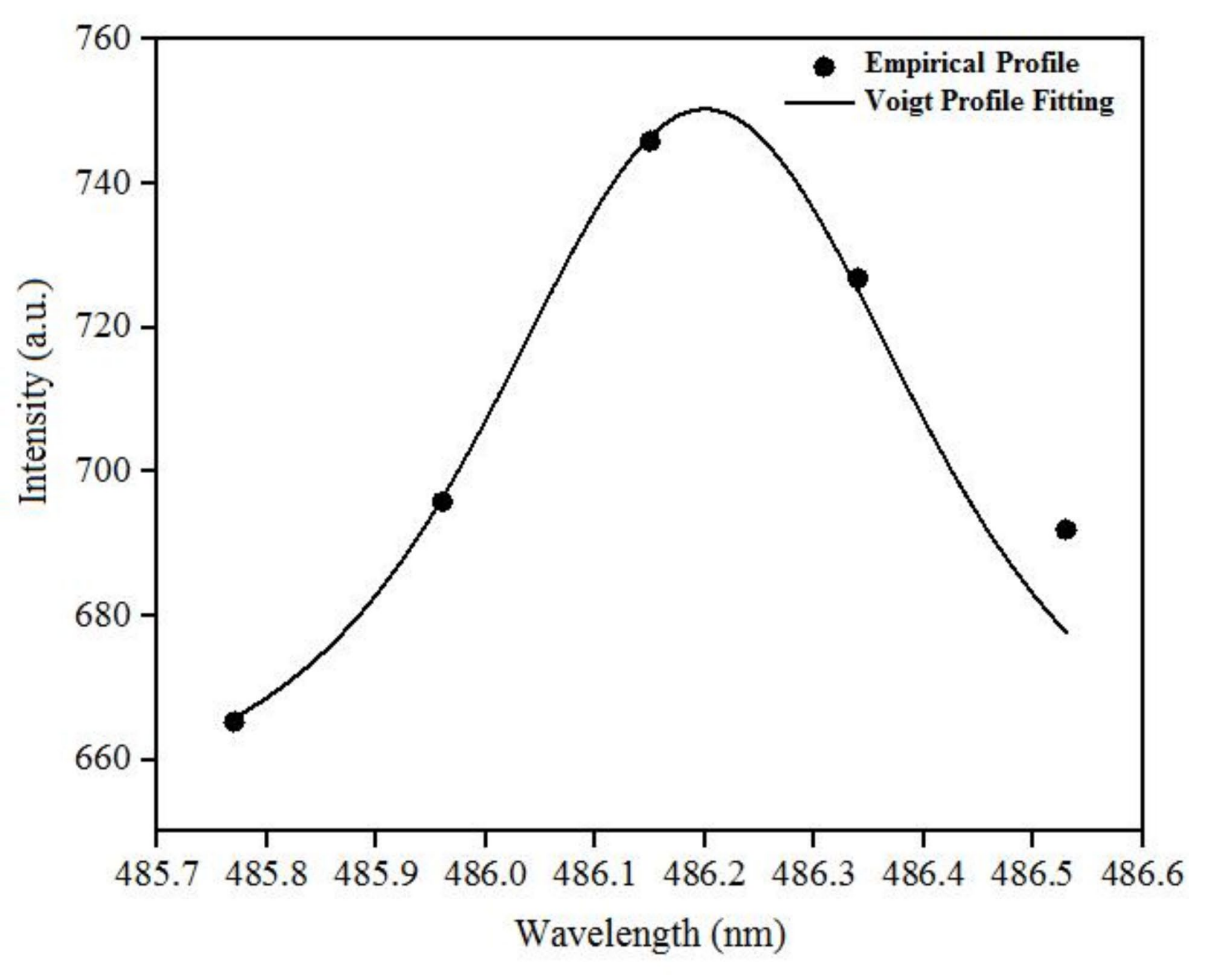
The typical Voigt-function fitting of the $\:{\text{H}}_{\beta\:}$ empirical profile of N I spectral lines at 7 kV.

The plasma electron density, $\:1.3330\times\:{10}^{21}{\:\text{m}}^{-3}$, was calculated by Stark broadening of the hydrogen Balmer $\:{\text{H}}_{\beta\:}$spectral line and analyzing the full-width at half-maximum (FWHM). The FWHM of the Stark broadening relating to the electron density is described by Eq. (2)^45–47^

$$\:\varDelta\:{{\uplambda\:}}_{\text{s}\text{t}\text{a}\text{r}\text{k}}=\varDelta\:{{\uplambda\:}}_{\text{L}\text{o}\text{r}\text{e}\text{n}\text{t}\text{z}}-\left(3.6\right)\left(\frac{\text{P}\left(\text{a}\text{t}\text{m}\right)}{{{\text{T}}_{\text{g}}}^{0.7}\left(\text{K}\right)}\right)=4.8\left(\text{n}\text{m}\right){\left(\frac{{\text{n}}_{\text{e}}\left({\text{m}}^{-3}\right)}{{10}^{23}\left({\text{m}}^{-3}\right)}\right)}^{0.68116}\:\:\:\:\:\:\:\:\:\:\left(2\right)$$

where T = 300 K and P = 1 atm. Figure 3 shows the typical Voigt-function fitting of the $\:{\text{H}}_{\beta\:}$ empirical profile at 7 kV.

## ORP and pH changes of tap water by cathodic plasma electrolysis

**Fig. 4:**
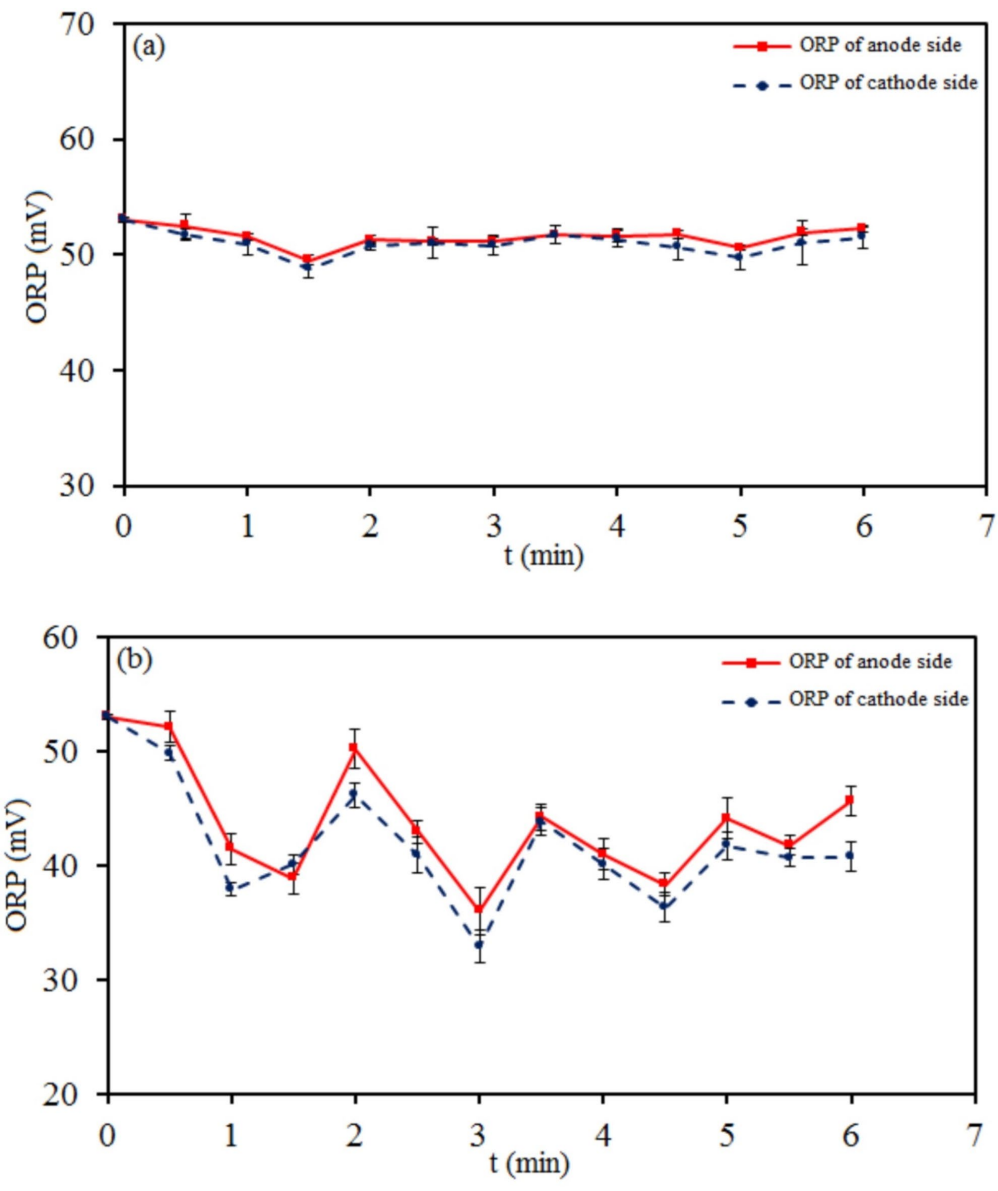
ORP-time plots of tap water in two modes of cathodic plasma electrolysis with (**a**) A and (**b**) B connectors.

The pH and ORP are two dependent indicators used in the water treatment^48,49^. The oxidation-reduction potential of water is an important indicator of the pollution degree, which determines the potential rate to carry out the reaction and accept and donate electrons^50,51^. ORP is a general measure of the oxidative stress in a biological system. Redox potential governs the transformation of organic compounds in water. In this study, a functional green method was used to control the ORP of the water through the composition of two galvanic and electrolytic cells by cold atmospheric plasma. A high-voltage direct current power supply was used for plasma production at 7 kV. The changes were small for ORP (and pH) at voltages less than 7 kV. The high electron temperature and reactive environment generated by NTP led to the high efficiency of liquid-phase chemical reactions such as treating water. Figure 4 shows the results of ORP-time changes of tap water in cathodic plasma electrolysis with (A) and (B) connectors. According to Fig. 4, the water ORP fluctuated during electrolysis on both the anode half-cell and the cathode half-cell at (A) and (B) connectors. It was positive and changed partially in (A) and significantly in (B). The electric potential energy was not higher than before plasma electrolysis and changes were almost similar on both sides. Both sides reacted as a single cell containing water without any connector. In other words, the water became acidic or alkaline in both half-cells simultaneously, which resulted in the same ORP changes in the two half-cells concurrently.

**Fig. 5:**
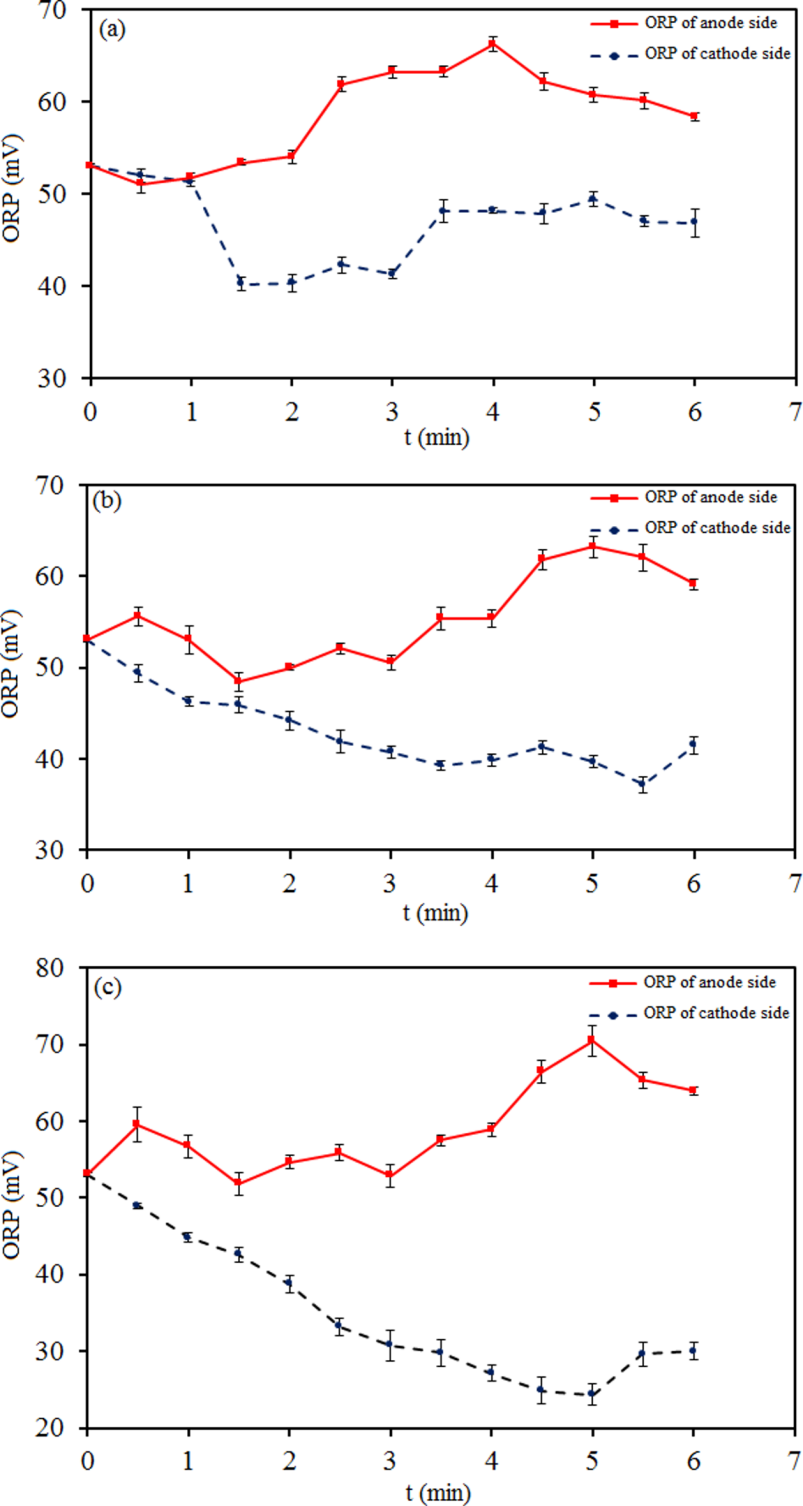
ORP-time plots of tap water in three modes of cathodic plasma electrolysis with (**a**) C, (**b**) D, and (**c**) E connectors.

Figure 5 demonstrates the results of ORP-time changes of tap water in cathodic plasma electrolysis with (C), (D), and (E) connectors. According to Fig. 5, the changes of water ORP were obtained approximately inverse during electrolysis on the anode half-cell and the cathode half-cell at (C), (D), and (E) connectors. The electric potential energy was almost higher on the anode half-cell and lower on the cathode half-cell than before plasma electrolysis.

Figures 4 and 5 show the oxidation power was dominant over the reduction power in the redox reactions of all setups. It was greater in the anode half-cells of (C), (D), and (E) connectors than in the others. In (C), (D), and (E) modes, the species tended to be oxidized as reducers on the cathode side and reduced as oxidizers on the anode side. In other words, comparing the two sides shows water has more anti-oxidation characteristics on the cathode side and more oxidizing potential on the anode side. In (A) and (B) modes, the changes in the reducing and oxidizing agents were the same on both half-cells and it was impossible to control them differently from each other.

**Fig. 6:**
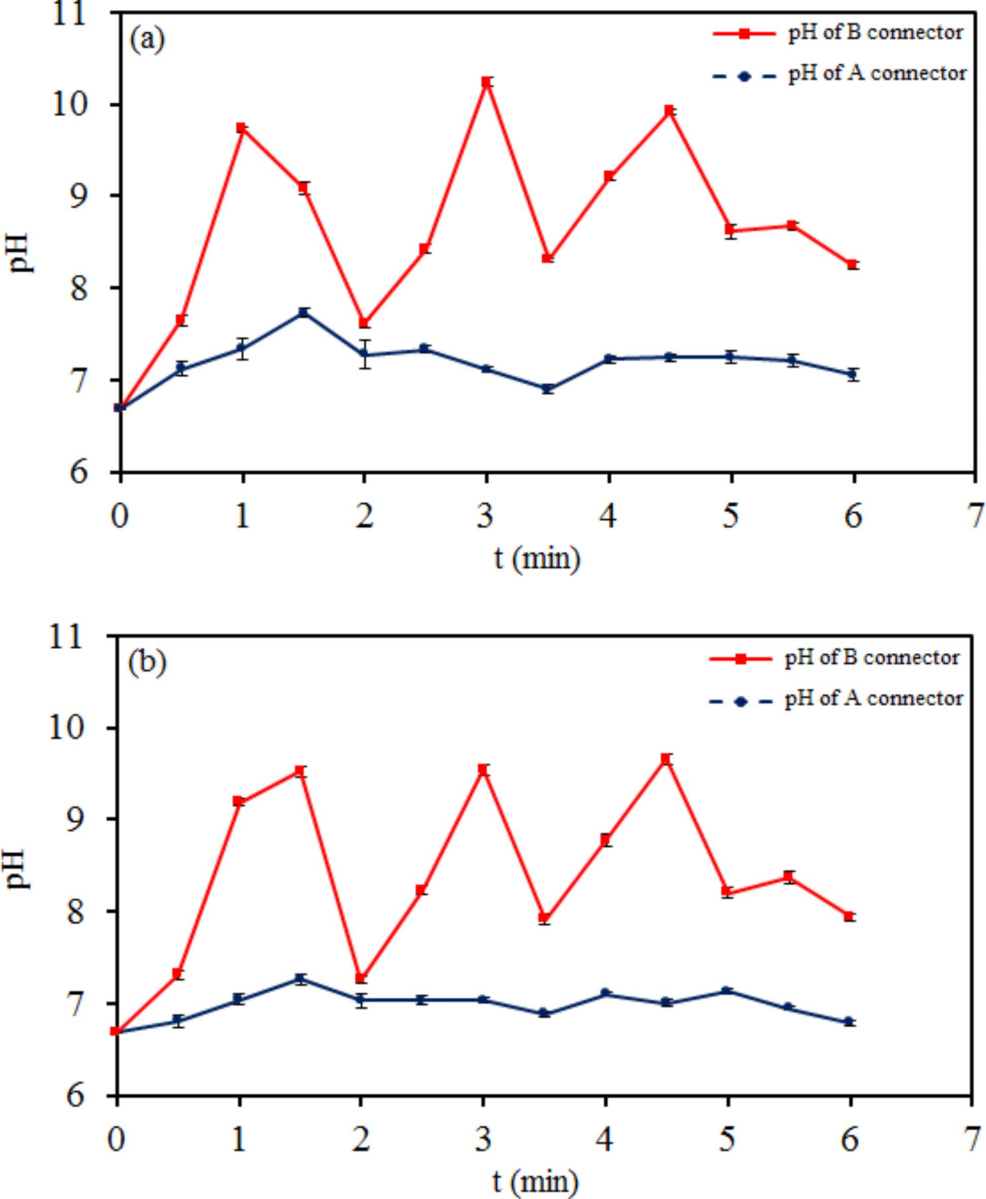
pH-time plots of tap water in two modes of cathodic plasma electrolysis with A and B connectors on (**a**) the anode side and (**b**) the cathode side.

**Fig. 7:**
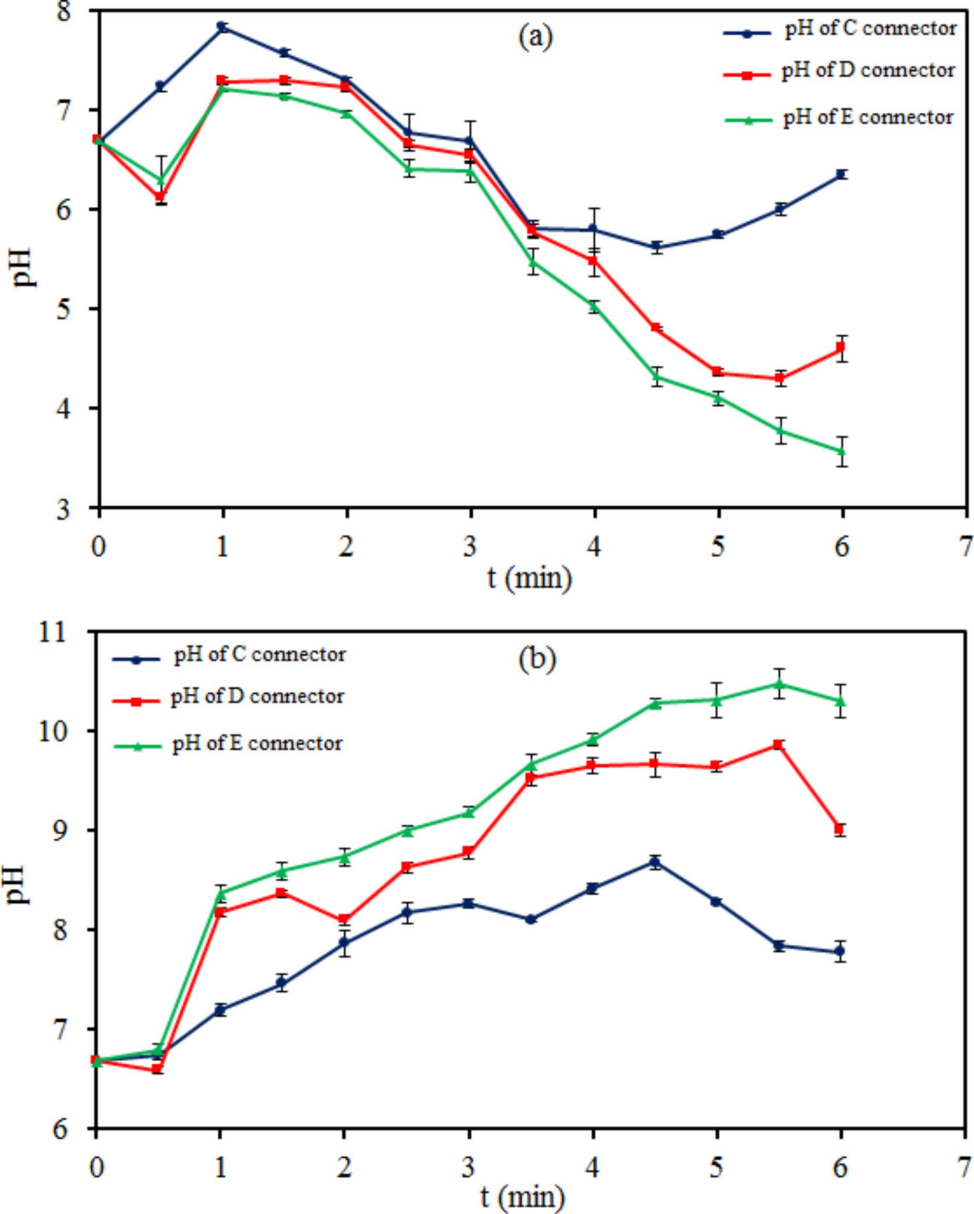
pH-time plots of tap water in three modes of cathodic plasma electrolysis with C, D, and E connectors on (**a**) the anode side and (**b**) the cathode side.

Figures 6 and 7 demonstrate the results of pH-time changes of tap water in cathodic plasma electrolysis with (A), (B), (C), (D), and (E) connectors. The changes in pH were obtained approximately inverse of ORP during electrolysis on the half-cells at all connectors. The hydrogen and hydroxide ions concentration $\:\left({[\text{H}}^{+}\left(\text{a}\text{q}\right)\right]={10}^{-\text{p}\text{H}},\:\left[{\text{O}\text{H}}^{-}\right(\text{a}\text{q}\left)\right]={10}^{-\text{p}\text{O}\text{H}})$ changes were the same and close to each other on the half-cells at (A) and (B) connectors. It was different for the anodic and cathodic half-cells at (C), (D), and (E) connectors.

**Fig. 8:**
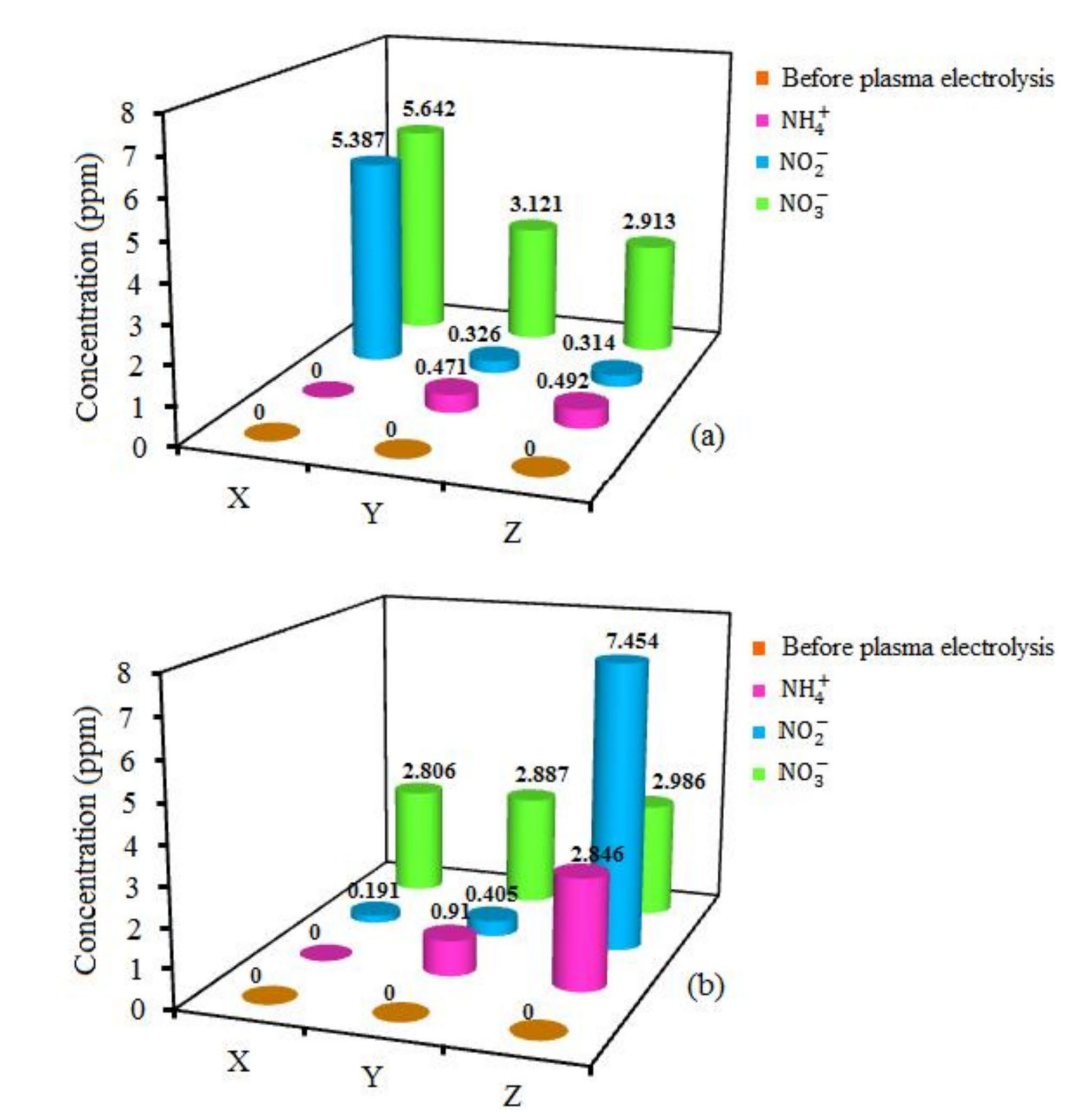
The concentrations of ammonium, nitrite, and nitrate ions synthesized by cathodic plasma electrolysis (**a**) on the anode side and (**b**) on the cathode side (X: with water, Y: with 20% agar solution, and Z: 30% agar solution).

Many investigations have been reported on the concentration change of nitrogen species like ammonia, nitrite, and nitrate in water by interaction with plasma. These reports were often about single-chamber reactors with acidic or alkaline properties with positive or negative ORP, respectively^52–55^. Due to the lack of information about the changes in the concentration of nitrogen species in two-chamber reactors with simultaneously acidic and basic properties, we focused on clarifying this aspect of plasma interaction with water and its role in nitrification. According to the results obtained from the connectors of the plasma electrolysis reactor, the reactor with a wide connector was chosen to continue the investigation.

## Plasma nitrification

By starting the discharge, not only did the current of the external source participate in chemical reactions, but also the electrons and ions of atmospheric plasma. $\:\text{N}{\text{O}}_{\text{x}}$species were produced through various reactions by nitrogen plasma formation and contact with the water surface^56^. The two-stage plasma nitrification occurred due to the production of plasma $\:\text{N}{\text{O}}_{\text{x}}$species in water. The first stage was the oxidation of ammonia to nitrite. At this stage, ammonia was synthesized by nitrogen gas fixation through plasma electrolysis in water^57^. Plasma electrolysis was done using nitrogen atmospheric pressure plasma with DC discharge interacting with the water surface. In the second stage, the resulting nitrite was oxidized to nitrate. The concentrations of ammonium $\:\left(\text{N}{\text{H}}_{4}^{+}\right)$ cation, nitrite $\:\left(\text{N}{\text{O}}_{2}^{-}\right)$, and nitrate $\:\left(\text{N}{\text{O}}_{3}^{-}\right)$ anions before and after plasma electrolysis at the anode and cathode sides with the wide connector filled with tap water and agar (20% and 30%) solutions are shown in Fig. 8. There was no $\:\text{N}{\text{H}}_{4}^{+}$, $\:\text{N}{\text{O}}_{2}^{-}$, and $\:\text{N}{\text{O}}_{3}^{-}$ before plasma electrolysis inside the tap water.

**Fig. 9:**
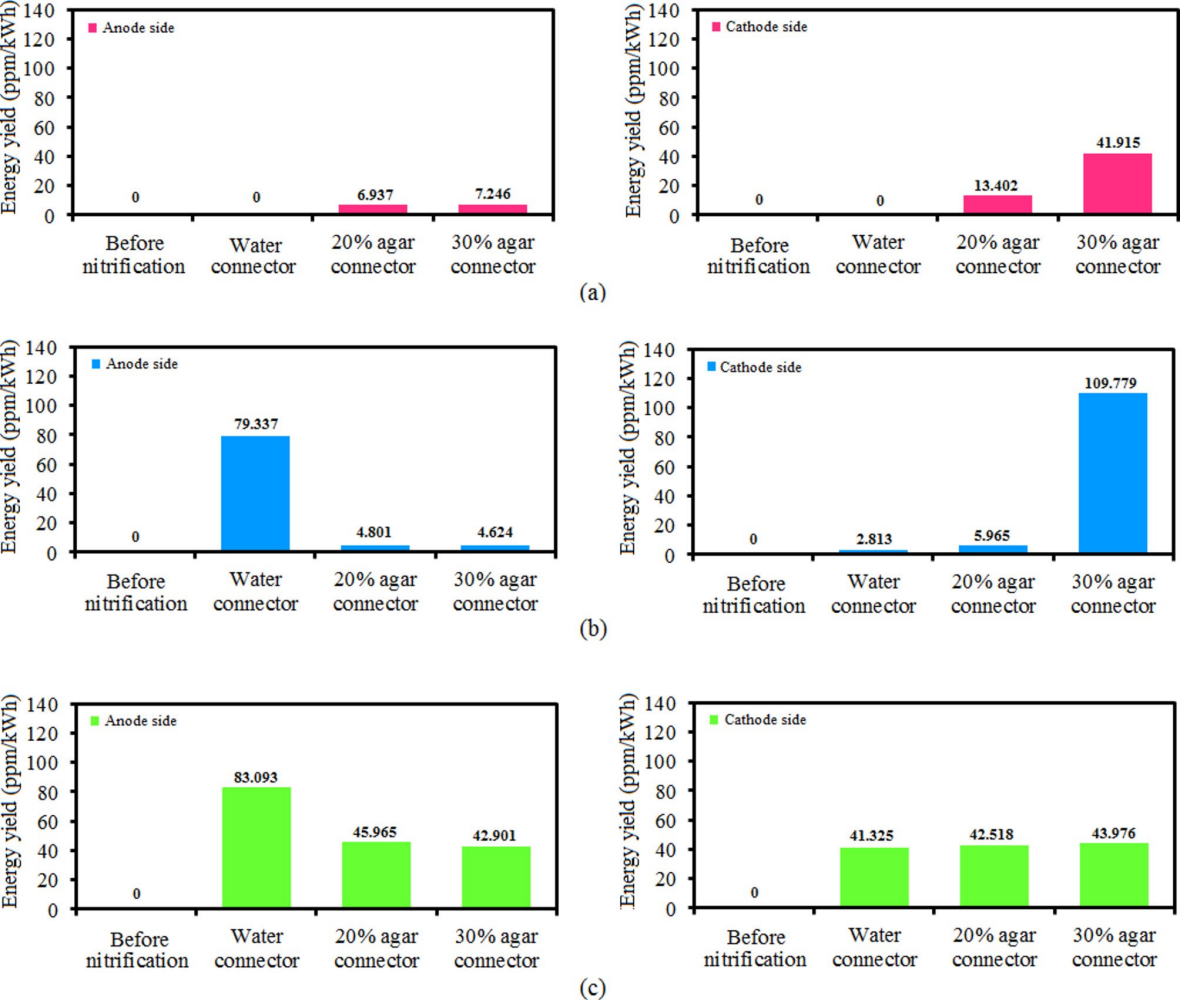
The synthesis energy yield of (**a**) ammonium, (**b**) nitrite, and (**c**) nitrate ions on the anode and cathode sides with water, 20% agar solution, and 30% agar solution connectors.

The concentrations of ions were obtained ($$\:\text{N}{\text{H}}_{4}^{+}$$: 0 ppm, 0.471 ppm, 0.492 ppm), ($$\:\text{N}{\text{O}}_{2}^{-}$$: 5.387 ppm, 0.326 ppm, and 0.314 ppm), and ($$\:\text{N}{\text{O}}_{3}^{-}$$: 5.642 ppm, 3.121ppm, 2.913 ppm) on the anode side with water, 20% agar, and 30% agar connector, respectively. The concentrations of ions were measured ($$\:\text{N}{\text{H}}_{4}^{+}$$: 0 ppm, 0.910 ppm, 2.846 ppm), ($$\:\text{N}{\text{O}}_{2}^{-}$$: 0.191 ppm, 0.405 ppm, and 7.454 ppm), and ($$\:\text{N}{\text{O}}_{3}^{-}$$: 2.806 ppm, 2.887 ppm, 2.986 ppm) on the cathode side with water, 20% agar, and 30% agar connector, respectively. Figure 9 shows the synthesis energy yield of ammonium, nitrite, and nitrate ions on the anode and cathode sides with water, 20% agar solution, and 30% agar solution connectors. The estimation of the reactor cost based on lab-scale equipment of this investigation stands at $2,500.

The reactions leading to ammonia, nitrite, and nitrate production by plasma formation are presented in the following. The reactions on the cathode side are denoted with C, and those on the anode side are marked with A. On the cathode side, nitrogen cation $\:{(\text{N}}^{+})$ is produced as a result of the dissociation ionization reaction of nitrogen gas $\:{(\text{N}}_{2})$, and hydrogen gas $\:{(\text{H}}_{2})$ and hydroxide anion $\:{(\text{O}\text{H}}^{-})$are also yielded by water plasma electrolysis^58,59^. The ion-molecule reaction of $\:{\text{N}}^{+}$ and $\:{\text{H}}_{2}$ in water causes the synthesis of ammonium $\:\left(\text{N}{\text{H}}_{4}^{+}\right)$ which would react with $\:{\text{O}\text{H}}^{-}$ and produce ammonia $\:\text{N}{\text{H}}_{3}$in water at the cathode side^57,60^.

$$\:{\text{N}}_{2}+{\text{e}}^{-}\to\:{\text{N}}^{+}+\text{N}+{2\text{e}}^{-}\:\:\:\:\:\left(\text{C}\right)\:\:\:\:\:\:\:\:\:\:\:\:\:\:\:\:\:\:\:\:\:\:\:\:\:\:\:\:\:\:\:\:\:\:\:\:\:\:\:\:\:\:\:\:\:\:\:\:\:\:\:\:\:\:\:\:\:\:\:\:\:\:\:\:\:\:\:\:\:\:\:\:\:\:\:\:\:\:\:\:\:\:\:\:\:\:\:\:\:\:\left(1\right)$$

$$\:{2\text{H}}_{2}\text{O}+2{\text{e}}^{-}\to\:{\text{H}}_{2}+{2\text{O}\text{H}}^{-}\:\:\left(\text{C}\right)\:\:\:\:\:\:\:\:\:\:\:\:\:\:\:\:\:\:\:\:\:\:\:\:\:\:\:\:\:\:\:\:\:\:\:\:\:\:\:\:\:\:\:\:\:\:\:\:\:\:\:\:\:\:\:\:\:\:\:\:\:\:\:\:\:\:\:\:\:\:\:\:\:\:\:\:\:\:\:\:\:\:\:\:\:\:\:\:\:\:\left(2\right)$$

$$\:{\text{N}}^{+}+{\text{H}}_{2}\to\:\text{N}{\text{H}}^{+}+\text{H}\:\:\:\:\:\:\:\:\:\:\:\:\:\left(\text{C}\right)\:\:\:\:\:\:\:\:\:\:\:\:\:\:\:\:\:\:\:\:\:\:\:\:\:\:\:\:\:\:\:\:\:\:\:\:\:\:\:\:\:\:\:\:\:\:\:\:\:\:\:\:\:\:\:\:\:\:\:\:\:\:\:\:\:\:\:\:\:\:\:\:\:\:\:\:\:\:\:\:\:\:\:\:\:\:\:\:\:\:\:\left(3\right)$$

$$\:{\text{N}\text{H}}^{+}+{\text{H}}_{2}\to\:\text{N}{\text{H}}_{2}^{+}+\text{H}\:\:\:\:\:\:\:\:\:\left(\text{C}\right)\:\:\:\:\:\:\:\:\:\:\:\:\:\:\:\:\:\:\:\:\:\:\:\:\:\:\:\:\:\:\:\:\:\:\:\:\:\:\:\:\:\:\:\:\:\:\:\:\:\:\:\:\:\:\:\:\:\:\:\:\:\:\:\:\:\:\:\:\:\:\:\:\:\:\:\:\:\:\:\:\:\:\:\:\:\:\:\:\:\:\:\left(4\right)$$

$$\:\text{N}{\text{H}}_{2}^{+}+{\text{H}}_{2}\to\:\text{N}{\text{H}}_{3}^{+}+\text{H}\:\:\:\:\:\:\:\:\:\left(\text{C}\right)\:\:\:\:\:\:\:\:\:\:\:\:\:\:\:\:\:\:\:\:\:\:\:\:\:\:\:\:\:\:\:\:\:\:\:\:\:\:\:\:\:\:\:\:\:\:\:\:\:\:\:\:\:\:\:\:\:\:\:\:\:\:\:\:\:\:\:\:\:\:\:\:\:\:\:\:\:\:\:\:\:\:\:\:\:\:\:\:\:\:\:\left(5\right)$$

$$\:\text{N}{\text{H}}_{3}^{+}+{\text{H}}_{2}\to\:\text{N}{\text{H}}_{4}^{+}+\text{H}\:\:\:\:\:\:\:\:\:\left(\text{C}\right)\:\:\:\:\:\:\:\:\:\:\:\:\:\:\:\:\:\:\:\:\:\:\:\:\:\:\:\:\:\:\:\:\:\:\:\:\:\:\:\:\:\:\:\:\:\:\:\:\:\:\:\:\:\:\:\:\:\:\:\:\:\:\:\:\:\:\:\:\:\:\:\:\:\:\:\:\:\:\:\:\:\:\:\:\:\:\:\:\:\:\:\left(6\right)$$

$$\:\text{N}{\text{H}}_{4}^{+}+\text{O}{\text{H}}^{-}\to\:\text{N}{\text{H}}_{3}+2{\text{H}}_{2}\text{O}\:\:\:\:\left(\text{C}\right)\:\:\:\:\:\:\:\:\:\:\:\:\:\:\:\:\:\:\:\:\:\:\:\:\:\:\:\:\:\:\:\:\:\:\:\:\:\:\:\:\:\:\:\:\:\:\:\:\:\:\:\:\:\:\:\:\:\:\:\:\:\:\:\:\:\:\:\:\:\:\:\:\:\:\:\:\:\:\:\:\:\:\:\:\:\:\left(7\right)$$

In the first stage of plasma nitrification (ammonia oxidizer), $\:\text{N}{\text{H}}_{3}$ reacts with hydroxide anion on the cathode side, $\:\text{N}{\text{H}}_{4}^{+}$obtained from the cathode reacts with oxygen on the anode side and nitrite anion is produced^61,62^.

$$\:\text{N}{\text{H}}_{3}+7{\text{O}\text{H}}^{-}\to\:\text{N}{\text{O}}_{2}^{-}+5{\text{H}}_{2}\text{O}+6{\text{e}}^{-}\:\:\:\:\:\:\:\left(\text{C}\right)\:\:\:\:\:\:\:\:\:\:\:\:\:\:\:\:\:\:\:\:\:\:\:\:\:\:\:\:\:\:\:\:\:\:\:\:\:\:\:\:\:\:\:\:\:\:\:\:\:\:\:\:\:\:\:\:\:\:\:\:\:\:\:\:\:\:\:\:\left(8\right)$$

$$\:2\text{N}{\text{H}}_{4}^{+}+3{\text{O}}_{2}\to\:2\text{N}{\text{O}}_{2}^{-}+4{\text{H}}^{+}+2{\text{H}}_{2}\text{O}\:\:\:\:\left(\text{A}\right)\:\:\:\:\:\:\:\:\:\:\:\:\:\:\:\:\:\:\:\:\:\:\:\:\:\:\:\:\:\:\:\:\:\:\:\:\:\:\:\:\:\:\:\:\:\:\:\:\:\:\:\:\:\:\:\:\:\:\:\:\:\:\:\:\:\:\:\:\left(9\right)$$

Ammonia without charge (reached from the cathode side) reaction with oxygen and electron might also lead to hydroxylamine $\:\left(\text{N}{\text{H}}_{2}\text{O}\text{H}\right)$on the anode side with the connector C. Hydroxylamine reacts with water and synthesizes nitrite^63^.

$$\:\text{N}{\text{H}}_{3}+{\text{O}}_{2}+2{\text{e}}^{-}\to\:\text{N}{\text{H}}_{2}\text{O}\text{H}+{\text{H}}_{2}\text{O}\:\:\:\:\:\:\:\:\:\:\left(\text{A}\right)\:\:\:\:\:\:\:\:\:\:\:\:\:\:\:\:\:\:\:\:\:\:\:\:\:\:\:\:\:\:\:\:\:\:\:\:\:\:\:\:\:\:\:\:\:\:\:\:\:\:\:\:\:\:\:\:\:\:\:\:\:\:\:\:\:\left(10\right)$$

$$\:\text{N}{\text{H}}_{2}\text{O}\text{H}+{\text{H}}_{2}\text{O}\to\:\text{N}{\text{O}}_{2}^{-}+5{\text{H}}^{+}+4{\text{e}}^{-}\:\:\:\:\:\:\left(\text{A}\right)\:\:\:\:\:\:\:\:\:\:\:\:\:\:\:\:\:\:\:\:\:\:\:\:\:\:\:\:\:\:\:\:\:\:\:\:\:\:\:\:\:\:\:\:\:\:\:\:\:\:\:\:\:\:\:\:\:\:\:\:\:\:\:\:\:\:\left(11\right)$$

Nitrite might be transferred to the anode side or remain on the cathode side. Plasma electrolysis produces hydrogen cation $\:{(\text{H}}^{+})$ and oxygen gas $\:\left({\text{O}}_{2}\right)$on the anode side^57^. In the second stage of plasma nitrification (nitrite oxidizer), nitrite reacts with $\:{\text{H}}^{+}$ on both the anode and cathode sides (on the anode side, $\:{\text{H}}^{+}$ is produced from plasma electrolysis, on the cathode side, $\:{\text{H}}^{+}$ is reached from the anode side) and produces $\:\text{N}{\text{O}}_{3}^{-}$^64^. Furthermore, the resulting nitrite anion of the first stage reacts with $\:{\text{O}}_{2}$obtained from the plasma electrolysis of water on the anode side, and finally, nitrate anion is obtained^65^.

$$\:2{\text{H}}_{2}\text{O}\to\:{\text{O}}_{2}+{4\text{H}}^{+}+4{\text{e}}^{-}\:\:\:\:\left(\text{A}\right)\:\:\:\:\:\:\:\:\:\:\:\:\:\:\:\:\:\:\:\:\:\:\:\:\:\:\:\:\:\:\:\:\:\:\:\:\:\:\:\:\:\:\:\:\:\:\:\:\:\:\:\:\:\:\:\:\:\:\:\:\:\:\:\:\:\:\:\:\:\:\:\:\:\:\:\:\:\:\:\:\:\:\:\left(12\right)$$

$$\:3\text{N}{\text{O}}_{2}^{-}+3{\text{H}}^{+}\to\:\text{N}{\text{O}}_{3}^{-}+2\text{N}\text{O}+{\text{H}}_{3}{\text{O}}^{+}\:\:\:\:\:\left(\text{A}\right)\&\left(\text{C}\right)\:\:\:\:\:\:\:\:\:\:\:\:\:\:\:\:\:\:\:\:\:\:\:\:\:\:\:\:\:\:\:\:\:\:\:\:\:\:\:\:\:\:\:\:\:\:\:\:\:\:\:\:\left(13\right)$$

$$\:2\text{N}{\text{O}}_{2}^{-}+{\text{O}}_{2}\to\:2\text{N}{\text{O}}_{3}^{-}\:\:\:\:\:\:\:\:\:\:\:\:\:\left(\text{A}\right)\:\:\:\:\:\:\:\:\:\:\:\:\:\:\:\:\:\:\:\:\:\:\:\:\:\:\:\:\:\:\:\:\:\:\:\:\:\:\:\:\:\:\:\:\:\:\:\:\:\:\:\:\:\:\:\:\:\:\:\:\:\:\:\:\:\:\:\:\:\:\:\:\:\:\:\:\:\:\:\:\:\:\:\:\left(14\right)$$

On the other hand, the reaction of $\:{\text{H}}^{+}$ with $\:\text{N}{\text{O}}_{2}^{-}$ and $\:\text{N}{\text{O}}_{3}^{-}$ might reproduce an amount of $\:\text{N}{\text{H}}_{4}^{+}$on the anode side^61,65,66^.

$$\:\text{N}{\text{O}}_{2}^{-}+{8\text{H}}^{+}+6{\text{e}}^{-}\to\:\text{N}{\text{H}}_{4}^{+}+2{\text{H}}_{2}\text{O}\:\:\:\:\:\:\:\left(\text{A}\right)\:\:\:\:\:\:\:\:\:\:\:\:\:\:\:\:\:\:\:\:\:\:\:\:\:\:\:\:\:\:\:\:\:\:\:\:\:\:\:\:\:\:\:\:\:\:\:\:\:\:\:\:\:\:\:\:\:\:\:\:\:\:\:\left(15\right)$$

$$\:\text{N}{\text{O}}_{3}^{-}+{10\text{H}}^{+}+8{\text{e}}^{-}\to\:\text{N}{\text{H}}_{4}^{+}+3{\text{H}}_{2}\text{O}\:\:\:\:\:\left(\text{A}\right)\:\:\:\:\:\:\:\:\:\:\:\:\:\:\:\:\:\:\:\:\:\:\:\:\:\:\:\:\:\:\:\:\:\:\:\:\:\:\:\:\:\:\:\:\:\:\:\:\:\:\:\:\:\:\:\:\:\:\:\:\:\:\left(16\right)$$

Figure 8 shows a direct proportion between the concentration of $\:\text{N}{\text{O}}_{2}^{-}$ and $\:\text{N}{\text{O}}_{3}^{-}$ anions and $\:\text{N}{\text{H}}_{4}^{+}$ cation on the cathode side. That is the reverse on the anode side. Hydroxide anions transferring toward the anode side decrease in D and E connectors proportional to the agar concentration, and the concentration of $\:\text{N}{\text{H}}_{4}^{+}$ cation increases on the cathode side. Subsequently, the concentration of $\:\text{N}{\text{O}}_{2}^{-}$ and $\:\text{N}{\text{O}}_{3}^{-}$ anions also increases on the cathode side. The agar concentration of the connector also affects the ions concentration on the anode side. As the connector concentration increases, fewer nitrite and nitrate anions transmit to the anode. The ammonium concentration on the anode side increases due to the decreasing transfer to the cathode side and the growth of $\:{\text{H}}^{+}$ cations accumulation on the anode side. The results of pH in Fig. 7 and the concentration of ions in Fig. 8 demonstrate that increasing the concentration of $\:{\text{O}\text{H}}^{-}$ anion directly has a positive effect on the concentration of $\:\text{N}{\text{H}}_{4}^{+}$ cation, which causes a positive effect on $\:\text{N}{\text{O}}_{2}^{-}$ and $\:\text{N}{\text{O}}_{3}^{-}$ cations synthesis.

**Fig. 10:**
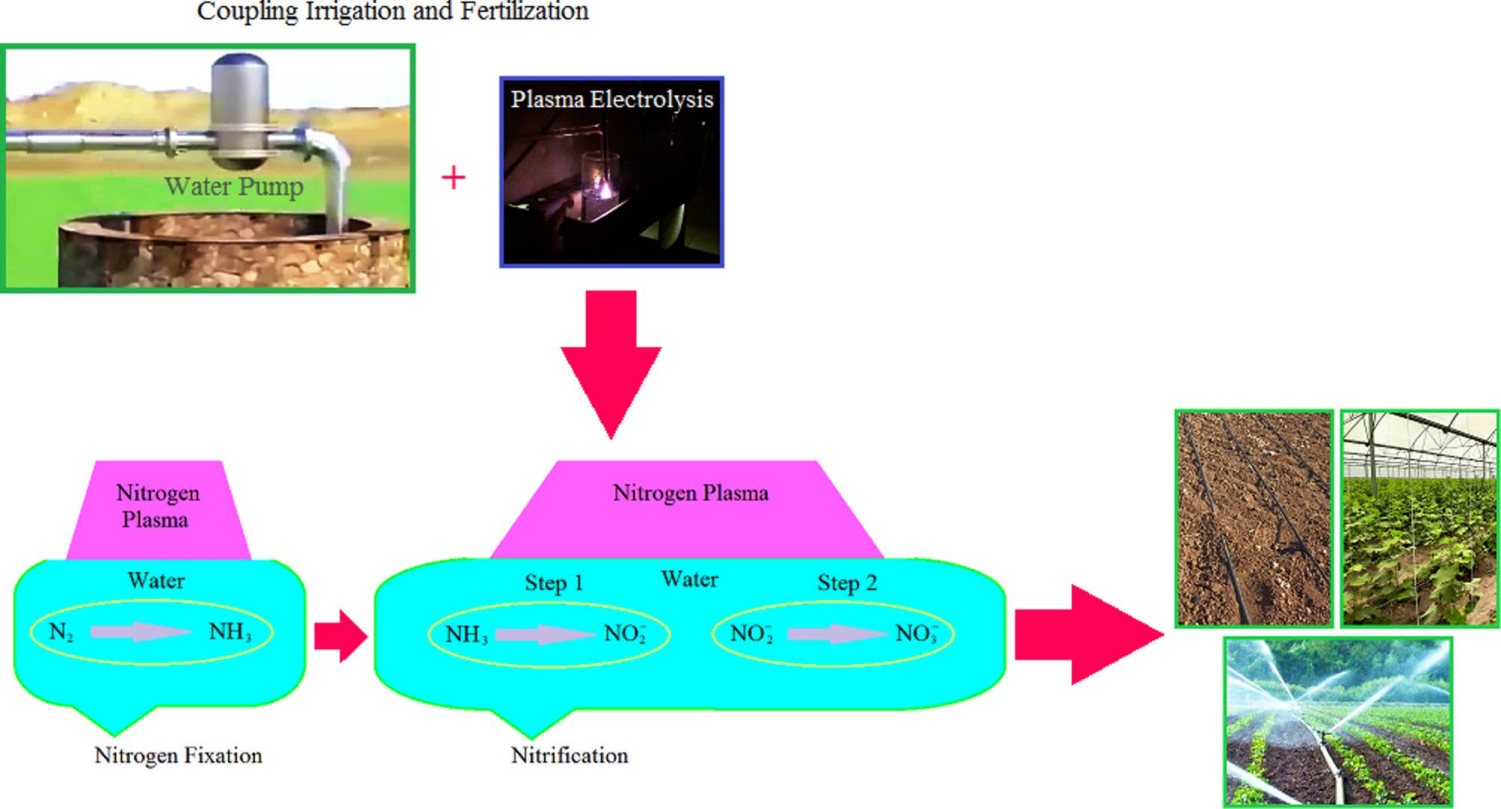
The designed reactor installed on the irrigation system of field for Coupling irrigation and fertilization.

## Discussion

Irrigation water quality is an evaluative aspect of crop production that depends on many factors including ORP of water. Redox potential is an essential indicator of the properties of water in controlling the water treatment process at purification facilities. Natural waters on the earth are usually determined by more positive redox potential because of contact with the atmosphere. In contrast, underground waters interacting with sulfides, silicates, and organic matter have less ORP than natural waters^67^. On the other hand, water with undesirable quality can lead to slow growth, bad appearance quality, and even the gradual death of plants^68,69^. Nitrogen is substantial to biological systems, because of its key role in biochemical reactions, and a sufficient supply of nitrogen species is required to achieve high productivity levels in agricultural fields^70^. Fertilization is one of the ways to solve this problem and improve crops by traditional methods, which can be affected by many factors, such as climatic and environmental conditions of cultivation, and may not be used optimally. For example, most fertilizers are lost as they flow or evaporate. Inadequate use of nutrients can destroy soil fertility and lead to degradation and poor soil productivity in the long term. On the other hand, excessive usage of fertilizers causes negative impacts on the environment and leads to economic losses for farmers. In addition to storing in crop plants, chemical fertilizer usage above the threshold level pollutes the groundwater through penetration into the soil. Nitrate-based fertilizers are the most valid and most efficient nitrogen source available. Ammonium and nitrate are the two main forms of inorganic nitrogen, important in trace element absorption in plants, and required for physiological and biochemical procedures in plants that directly absorb. Ammonium and nitrate ratio is effective in nitrogen metabolism^71^. Applying ammonium nitrate is more instrumental in plant biomass accumulation than applying urea and calcium nitrate^72^. Different $\:{\text{N}\text{H}}_{4}^{+}:{\text{N}\text{O}}_{3}^{-}$ratios can affect the promotion of plant growth, root development, nutrient element accumulation, raising enzyme activities, and quality of fruits^72,73^. Improving nitrogen use efficiency (NUE = N output/ N input) is significant for sustainable agriculture because it can help decrease nitrogen losses to the environment, develop crop yield, and minimize the need for excessive nitrogen fertilization^32,74^. According to the results and the interpretations expressed, a green practical method free of chemicals is creatively suggested. The existence of a system that can produce different $\:{\text{N}\text{H}}_{4}^{+}:{\text{N}\text{O}}_{3}^{-}$ ratios and modify all kinds of water with various ORPs to enable water to irrigate and fertilize fields with diverse crops can be a cost-effective idea. To prevent the loss of nitrogen species effective at the growth of agricultural products, the suggested reactor can be used as an irrigation and fertilization combined system on farms and greenhouses either underground near the plant roots or on the ground (Fig. 10).

The designed reactor with two separate parts that create different properties in the redox potential (and pH) of water and at the same time enriches it with nitrogen forms effective in the growth of crops can be installed on the irrigation system of fields and greenhouses and can be used proportional to the environmental conditions and the type of crop. By managing the water modification in the desired reactor by the plasma electrolysis, the consumption of water and fertilizer and the resulting costs are saved, the side effects of excessive consumption of fertilizer are avoided, and clean products free of nutrient pollution are produced.

## Conclusion

In this research, a method to control the oxidation-reduction potential and pH of the water via cold atmospheric plasma electrolysis was investigated by the composition of galvanic and electrolytic cells. ORP and pH changes in tap water were measured and studied after plasma electrolysis in various configurations at different times. Those were different depending on the connector dimensions of the half-cells and connector filler. In the connector with large dimensions, ORP and pH increasing and decreasing for the anodic and the cathodic half-cells were observed reverse during different times, while these changes were obtained the same for both half-cells in the connector with small dimensions. In the wide connector, the species tended to be oxidized as reducers on the cathode side and reduced as oxidizers on the anode side. This process was the same on both sides at the same time in the narrow connector. According to the mentioned cases, the wide connector was chosen to investigate the process of plasma nitrification by plasma electrolysis in the first stages of plasma and species production. The results showed that there is a possibility of nitrification by plasma electrolysis, and the concentration of the main species involved in this process (ammonia/ammonium, nitrite, and nitrate) can change according to the type and concentration of connector filler. Furthermore, it has a direct relationship between $\:{\text{N}\text{H}}_{4}^{+}$ and hydroxide anions synthesis which also causes a direct effect on nitrite and nitrate cations synthesis. So, this plasma nitrification process can be used in agriculture, where it determines the availability of fertilizer nitrogen, and in wastewater treatment systems to complete the wastewater treatment process without the need for any chemicals. The introduced method can be replaced as an eco-friendly alternative to promote fertility while ensuring agricultural biosafety without any negative outcome on soil microflora. It is possible to save lots of water treatment and agriculture costs with the proposed reactor. The suggested setup can be one green advanced practice with variable-rate application of ammonium and nitrate on fields and greenhouses for the production of fruits, vegetables, flowers, and any other plants that require a special rate.

## Data Availability

The data sets generated during and/or analyzed during the current study are available in the OSF repository, [https://doi.org/10.17605/OSF.IO/U7KSA].
